# Laparoscopic Approach to Median Arcuate Ligament Syndrome: A Single-Center Experience

**DOI:** 10.3390/medicina62020356

**Published:** 2026-02-11

**Authors:** Matas Pažusis, Ieva Ramanauskaitė, Laima Maleckienė, Elita Drobužaitė, Linas Velička, Almantas Maleckas, Mindaugas Kiudelis

**Affiliations:** 1 Department of Surgery, Medical Academy, Lithuanian University of Health Science, 44307 Kaunas, Lithuania; matas.pazusis@lsmu.lt (M.P.); ieva.ramanauskaite@lsmuni.lt (I.R.); mindaugas.kiudelis@lsmu.lt (M.K.); 2 Department of Obstetrics and Gynaecology, Lithuanian University of Health Science, 44307 Kaunas, Lithuania; laima.maleckiene@lsmu.lt; 3 Department of Cardiac, Thoracic and Vascular Surgery, Medical Academy, Lithuanian University of Health Science, 44307 Kaunas, Lithuania; elita.drobuzaite@lsmu.lt (E.D.); linas.velicka@lsmu.lt (L.V.); 4 Department of Gastrosurgical Research and Education, Sahlgrenska Academy, University of Gothenburg, 405 30 Gothenburg, Sweden

**Keywords:** Dunbar syndrome, MALS, median arcuate ligament syndrome, laparoscopic decompression

## Abstract

*Background and Objectives*: Median arcuate ligament syndrome (MALS), also known as Dunbar syndrome, is a vascular compression disorder. Over time, laparoscopy has become increasingly important in the treatment of MALS, gradually replacing open surgical reconstruction as the preferred first-line approach in most cases. We present nine years of experience managing patients with MALS, aiming to contribute to the evidence supporting this long-debated condition. *Materials and Methods*: A single-center prospective observational cohort study analysis was conducted. CT angiography was used to confirm the diagnosis of MALS in all patients. All surgical patients completed the Gastrointestinal Quality of Life Index (GIQLI) and Gastrointestinal Symptom Rating Scale (GSRS) questionnaires preoperatively and postoperatively. All the patients underwent laparoscopic decompression. Postoperative follow-up visits were scheduled at one month and three years postoperatively. *Results*: During the study period, 37 patients were diagnosed with MALS, of whom 11 (29.7%) were symptomatic and underwent laparoscopic decompression. The operated cohort consisted of nine women and two men (mean age 56.7 ± 10.7 years). All patients reported postprandial epigastric pain, and 63.6% experienced weight loss. Laparoscopic decompression was successfully completed in all cases without intraoperative complications. Two patients had stenting after surgery, and in two, prior to surgery. The mean operative time was 103 ± 54 min, and the mean hospital stay was 4.2 ± 2.2 days. At 3-year follow-up, 36.4% of patients reported recurrent symptoms associated with recurrent celiac trunk stenosis on CT angiography. Overall, the patients had less indigestion and less pain; however, the differences did not reach a statistically significant level. *Conclusions*: Laparoscopic decompression of the median arcuate ligament is a feasible and safe treatment for carefully selected patients with symptomatic MALS, offering durable symptom relief in most cases with minimal morbidity.

## 1. Introduction

Median arcuate ligament syndrome (MALS), also known as Dunbar syndrome, is a vascular compression disorder. It occurs when the median arcuate ligament and the crura of the diaphragm compress the celiac artery. This condition was first described in 1917 by Lipschütz, who, upon conducting anatomical studies on cadaveric material, showed that the crura of the diaphragm can compress the celiac artery [1]. The median arcuate ligament is a fibrous band that anteriorly connects the crura of the diaphragm as they span the aortic orifice. A high origin of the celiac artery or a low insertion of the crura may predispose to the development of MALS. The degree of compression often increases during exhalation, as the upward displacement of the abdominal vessels, including the celiac trunk, brings it closer to the ligament. This compression may be caused by either thickened fibrous tissue or thin fibrous bands located in or near the orifice of the celiac artery [1].

The pathophysiology of MALS remains poorly understood, making its diagnosis difficult [2]. In approximately 10–24% of people, the celiac axis is located in close proximity to the median arcuate ligament and may be subject to compression, potentially leading to decreased blood flow to the organs it supplies and the development of clinical symptoms. According to Katz-Summercorn et al., in a study of 99 autopsies, 92.6% of cases demonstrated that the celiac trunk was located near the median arcuate ligament (without precise measurement of the distance), and in 33.7% of them, signs of its compression or deformation were found [3].

The incidence of median arcuate ligament syndrome is estimated at approximately 1–2 cases per 100,000 person-years. It most commonly occurs in young adults aged 20–40 years, predominantly in thin patients, with women being affected more frequently than men [4,5]. According to the European Society for Vascular Surgery guidelines for diseases of the mesenteric arteries and veins, MALS is considered the most common cause of isolated stenosis of one abdominal artery [6].

It should be noted that in 30–50% of cases, incidentally detected celiac trunk compression is asymptomatic [2]. Clinically, MALS typically presents with postprandial abdominal pain, nausea, vomiting, changes in eating behavior, and, over time, weight loss. Because these symptoms overlap with those of many more common diseases, diagnosis is often delayed [4]. Universally accepted diagnostic criteria for MALS are currently lacking. Many researchers believe that this disease often goes unrecognized, similar to chronic mesenteric ischemia, which is characterized by delays in diagnosis. This has led to a lack of large-scale clinical trials and has hampered the development of uniform diagnostic and treatment standards. As a result, most current recommendations are based on systematic reviews and meta-analyses, largely relying on individual clinical cases and small observational series [7]. Asymptomatic patients usually do not require invasive intervention, whereas in clinically overt MALS, surgical treatment is most often indicated. Surgery is the primary interventional option for MALS when symptoms and imaging indicate clinically significant celiac artery compression and alternative causes of pain have been ruled out. The key procedure is celiac axis decompression by dividing the median arcuate ligament and adjacent fibrous bands, often paired with celiac plexus/ganglion neurolysis to reduce a possible neuropathic pain component alongside vascular compression. It can be performed through an open approach, laparoscopically, or using robotic-assisted techniques [1,7]. If necessary, celiac artery bypass grafting or endovascular stenting are also considered.

Thus, in symptomatic patient cases, when stenosis of the celiac artery origin is related to extrinsic compression by the median arcuate ligament, endovascular stenting prior to decompression surgery should be generally contraindicated. Based on Rutherford’s *Vascular Surgery and Endovascular Therapy*, 10th edition, the recommended approach is open surgical or laparoscopic division of the median arcuate ligament followed by endovascular or open reconstruction of the celiac lesion [8]. Open vascular reconstruction may also be performed in severe, chronic lesions that need more durable anatomic correction [9]. Aorto-celiac trunk bypass is a major vascular surgery to create a new blood path around a blocked or narrowed celiac artery. This method is rarely applicable, only in cases of chronic arterial injury, which include heavy fibrosis, aneurysm formation, and failed prior less-invasive interventions. However, open vascular reconstruction should also be a second-stage treatment after primarily performing median arcuate ligament release [8].

Over time, the laparoscopic approach has become increasingly important in the treatment of MALS and has gradually replaced open surgical reconstruction in many cases, becoming the preferred first-line method [2]. Furthermore, small single-center studies have shown that laparoscopic release (section) of the median arcuate ligament may provide good short- and long-term results, with improvement or resolution of symptoms in 70–90% of patients [10]. Recent prospective and comparative cohort studies support laparoscopic MAL release as an effective minimally invasive approach in routine care, while highlighting the need to standardize operative technique and outcome reporting to enable better comparison across studies [4].

Robotic-assisted techniques have increasingly become popular in recent years, with the goal of enhancing dexterity and surgeon ergonomics during precise dissection at the celiac origin. Comparative and systematic evaluations indicate that robotic release is feasible and safe in experienced centers, with outcomes generally comparable to laparoscopy; however, cost, access, and appropriate patient selection remain key considerations [11,12,13,14,15]. An expanding technique-oriented literature, including detailed step-by-step laparoscopic descriptions, highlights ongoing efforts to standardize operative approach and minimize variability in outcomes [10,16].

On the one hand, an endovascular-only approach can manage patients’ symptoms and improve celiac artery patency for the short term [17]. On the other hand, external pressure leads to a higher risk of stent deformation, migration, or fracture, leading to restenosis and occlusion, thus increasing the risk for reintervention [18,19].

Reported rates of symptom relief vary substantially across studies, likely due to differences in diagnostic criteria, completeness of decompression, outcome definitions, and follow-up duration. A systematic review of treatment effectiveness underscored this heterogeneity and emphasized the need for standardized endpoints and longer-term follow-up [20]. Prognostic studies indicate that both patient-related factors and disease characteristics affect long-term outcomes, underscoring the need for careful patient selection and standardized postoperative evaluation [21]. Additional research on laparoscopic management of MALS is warranted because reported outcomes are highly inconsistent, likely reflecting differences in diagnostic standards, patient selection, surgical technique, and follow-up practices. Prospective cohorts using standardized imaging criteria and validated symptom and quality-of-life measures are needed to clarify the durability of clinical benefit and to identify when supplementary endovascular treatment is genuinely necessary [2,6,20,21].

In the present study, we outline nine years’ results in managing patients with MALS, aiming to analyze our institution’s experience in laparoscopic decompression, including preoperative assessment, surgical technique, postoperative outcomes, and patient-reported results.

## 2. Materials and Methods

This single-center prospective observational cohort included consecutive patients undergoing laparoscopic median arcuate ligament release for MALS at the Department of Surgery, Lithuanian University of Health Sciences. The surgeries were performed from January 2017 to December 2022, with a 3-year follow-up of all cases completed until December 2025. The study was approved by the Kaunas Regional Biomedical Research Ethics Committee (Protocol approval number BE-2-13). The study was conducted in accordance with the principles of the Helsinki Declaration. Written informed consent was obtained from all participants. All study data were deidentified prior to analysis.

Patients were included if they had clinically significant abdominal pain attributable to MALS (chronic postprandial epigastric/upper abdominal pain), CTA (Computed Tomography Angiography) evidence of celiac axis compression with characteristic morphology (“hooked” configuration) with or without post-stenotic dilatation, and celiac trunk stenosis ≥50% on CTA. All patients underwent standardized diagnostic evaluation, including laboratory blood tests, esophagogastroduodenoscopy, colonoscopy, and abdominal imaging (ultrasound, CT), to exclude other abdominal pathologies. Duplex ultrasound was not used as a mandatory inclusion criterion. CT angiography was used to confirm the diagnosis of MALS in all patients. CTA measurements were performed on multiplanar reconstructions, including sagittal views of the celiac axis. On CTA, celiac trunk stenosis was measured as the percentage reduction in vessel diameter at the site of maximal compression relative to a normal distal segment. In routine practice, CTA is typically acquired in end-inspiration. However, paired inspiratory/expiratory acquisitions were not routinely obtained, and therefore, respiratory variation was not systematically quantified. Thus, “hemodynamic significance” in this study was operationalized primarily by the magnitude of anatomic stenosis on CTA in the appropriate clinical context after exclusion of other etiologies. Images were reviewed by an experienced radiologist and verified by the surgical team. All patients were asked to complete the Gastrointestinal Quality of Life Index (GIQLI) and the Gastrointestinal Symptom Rating Scale (GSRS) preoperatively and again 3 years after surgery. The GIQLI total score ranges from 0 to 144, with higher scores indicating better quality of life, whereas GSRS domain scores range from 1 to 7, with higher scores reflecting more severe symptoms. In our study, all patients completed the questionnaires; no forms were excluded due to missing data.

**Figure 1:**
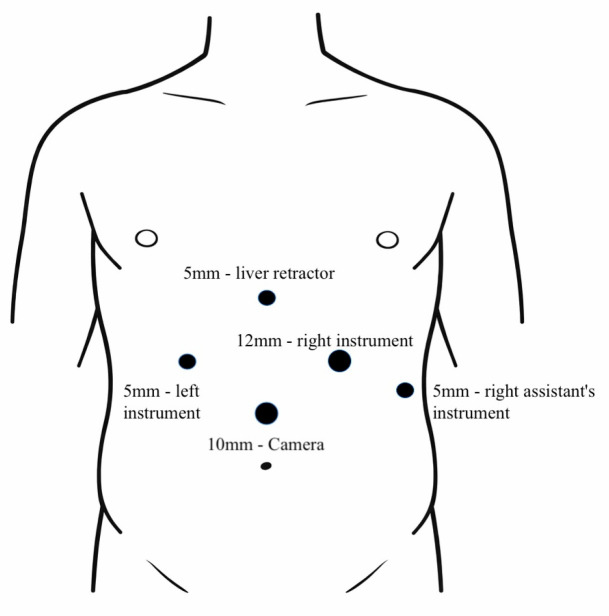
Trocar placement sites for laparoscopic release of median arcuate ligament.

**Figure 2:**
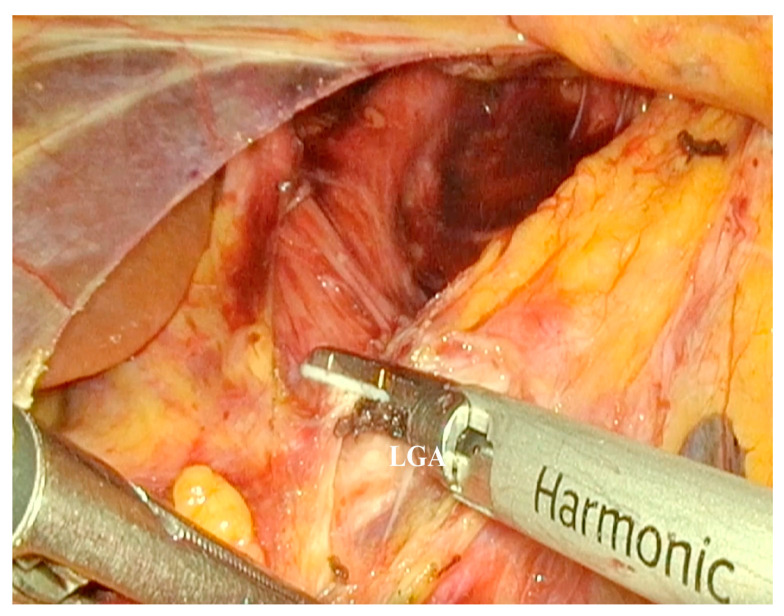
The tissues surrounding left gastric artery (LGA) were dissected to expose the site where the LGA originates from celiac trunk.

**Figure 3:**
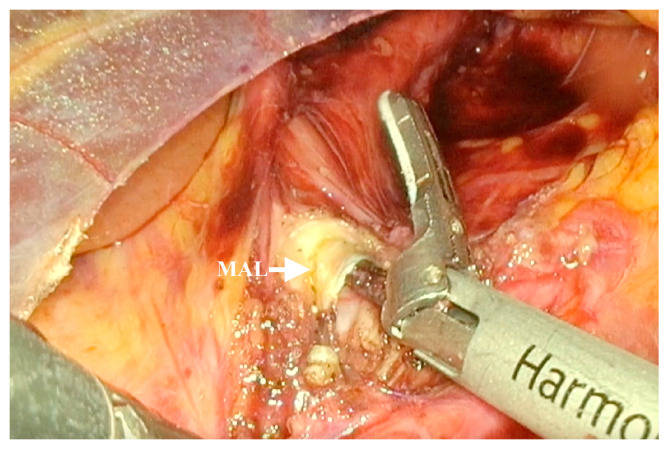
Dissection and division of the median arcuate ligament (MAL) were performed, achieving complete release of the celiac trunk.

**Figure 4:**
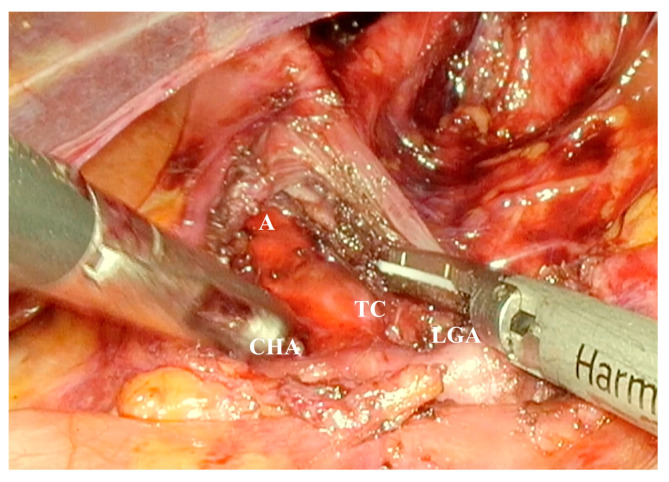
The celiac trunk was completely released, and its arterial surface was meticulously cleared of all compressive fibers of the median arcuate ligament. TC—celiac trunk; LGA—left gastric artery; CHA—common hepatic artery; A—aorta.

**Figure 5:**
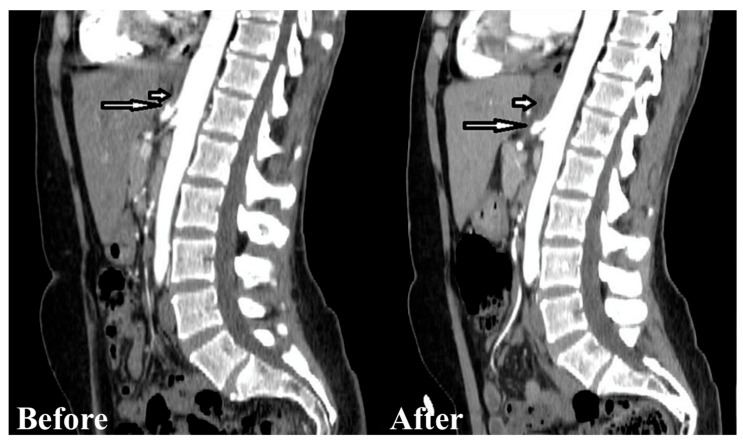
Before-surgery sagittal reconstruction of contrast-enhanced computed tomography demonstrates a short-segment stenosis of the celiac trunk with a characteristic hooked configuration and post-stenotic dilatation (long arrow). Thickening of the median arcuate ligament is also evident (short arrow). After-surgery sagittal computed tomography shows complete release of the celiac trunk stenosis (long arrow). Postoperative soft-tissue edema is visible in the surrounding tissues (short arrow).

The indications for laparoscopic surgery were clinically significant abdominal pain due to MALS, radiologically confirmed MALS on CT angiography, and celiac trunk stenosis of 50% or greater. The surgeries were performed by senior surgeons with extensive experience in laparoscopic surgery. The laparoscopic decompression was carried out using five trocars placed in the upper abdomen, following the trocar sites used in the laparoscopic fundoplication (Figure 1). A retractor was introduced in the subxiphoid region to medially lift the left lobe of the liver. Pneumoperitoneum was established at 12 mmHg. An ultrasonic dissector was used for tissue dissection and achieving hemostasis (Figure 2). Following the division of the gastrohepatic ligament, the common hepatic artery and the left gastric artery were dissected and found. Both arteries were traced to the origin of the celiac trunk, with no dissection of the splenic artery required. The right crus was dissected, and the median arcuate ligament was identified and divided using an energy device (Figure 3). Approximately 4–5 cm of the anterior surface of the aorta was exposed, and the celiac trunk was fully released. All fibrotic tissue and overlying nerve plexus on the celiac axis were meticulously removed (Figure 4). Intraoperative ultrasonography was not used to evaluate celiac artery flow following decompression. Intraoperative parameters recorded included operative time, estimated blood loss, and intraoperative complications. The effect of surgical release on celiac trunk patency was evaluated by postoperative CT angiography (Figure 5). Celiac artery stenting (adjunct endovascular treatment) was considered for patients with persistent, hemodynamically significant (>70%) residual stenosis on CT angiography after celiac decompression and, when indicated, was attempted using percutaneous transluminal angioplasty (PTA) followed by the placement of a balloon-expandable stent.

Postoperative follow-up after laparoscopic decompression for MALS was conducted in the outpatient clinic. Patients were evaluated for symptom relief, postoperative complications, and overall recovery, with additional visits scheduled as needed. Imaging with CT angiography was performed to assess celiac artery decompression and vascular patency. Patients attended outpatient follow-up visits one month after surgery and 3 years after surgery, with follow-up extended as necessary. Control CT angiography was performed in the first month postoperatively.

The collected data included age, gender, clinical symptom characteristics and diagnostic findings, American Society of Anesthesiologists (ASA) physical status classification, intraoperative findings and complications, postoperative complications and mortality, length of hospital stay, readmissions, and need for reintervention (including endovascular treatment). Complications were recorded and graded using Clavien-Dindo. Data were extracted from electronic medical records and study protocols, with values reported as mean ± standard deviation (SD).

The primary endpoint was clinical status at 3 years, defined as patient-reported presence/absence of MALS typical symptoms (postprandial epigastric pain/discomfort). Secondary endpoints included perioperative outcomes (technical success, complications, and length of stay), need for adjunct endovascular treatment (PTA/stenting), and CTA findings at follow-up (residual/recurrent stenosis). Recurrence was defined as the return of postprandial epigastric pain or discomfort after an initial improvement following surgery, as reported at the 3-year follow-up, regardless of whether reintervention was performed. Imaging findings were described qualitatively and were not required to meet any predefined criteria to be considered a recurrence. Clinical success was defined as a composite outcome including the resolution of MALS-related symptoms (postprandial epigastric/upper abdominal pain/discomfort) at follow-up, absence of recurrent hemodynamically relevant celiac artery stenosis on follow-up CT angiography, and improvement in nutritional status, assessed by postoperative weight regain and/or an increase in BMI compared with preoperative values.

Statistical analyses were conducted using the SPSS software package (version 21.0, SPSS Inc., Chicago, IL, USA). Numerical variables are presented as mean ± standard deviation (SD), while categorical variables are expressed as counts and percentages. Statistically significant differences were defined as *p* < 0.05, as determined using the Wilcoxon matched-pairs signed-rank test.

## 3. Results

During the study period, 37 patients were diagnosed with MALS (28 (75.6%) female and 9 (24.4%) male). Of these, only 11 patients (29.7%) had clinically significant symptoms consistent with MALS and hemodynamically relevant celiac trunk stenosis requiring laparoscopic decompression. The remaining 26 patients (70.3%) were diagnosed incidentally and, at the time of diagnosis, did not exhibit clinically meaningful symptoms compatible with MALS. They were informed about the diagnosis and referred to primary care for follow-up and were not included in the present study. Consequently, the development of interval symptoms could not be reliably determined in the nonoperative cohort.

Among the symptomatic patients who underwent laparoscopic decompression, nine (81.8%) were women and two (18.2%) were men, with ages ranging from 42 to 71 years (mean 56.7 ± 10.7 years). The duration of symptoms ranged from 2 months to 3 years. Intermittent epigastric pain was reported by all patients, and all the patients described postprandial exacerbation of the symptoms with relief during fasting. Seven patients (63.6%) experienced weight loss between 2 and 37 kg (mean 14 ± 11.6 kg). Mean BMI was 24.8 ± 7.3 kg/m^2^ prior to surgery. Physical examination consistently revealed epigastric tenderness without guarding or rebound. The demographic and clinical characteristics of patients are presented in Table 1.

The diagnosis of MALS was confirmed in all patients using CT angiography, which demonstrated high-grade stenosis of the anterior aspect of the proximal celiac axis due to extrinsic compression by the median arcuate ligament. Laparoscopic decompression of the median arcuate ligament was successfully completed in all patients without intraoperative complications. Two male patients had previously undergone unsuccessful celiac artery stenting prior to surgical referral, and two patients underwent postoperative stenting within one week after the laparoscopic decompression. Operative time ranged from 65 to 210 min, with a mean duration of 103 ± 54 min. Three of the patients had a history of previous upper abdominal surgery, but no conversions to open procedures were required. Postoperatively, patients were discharged between 1 and 9 days, with a mean hospital stay of 4.2 ± 2.2 days. The only postoperative complication was fever and laparoscopic trocar site infection accompanied by an elevated C-reactive protein level (253 mg/L) in one female patient, which resolved with antibiotic therapy (Clavien-Dindo Grade II). No hospital readmissions were reported. The summarized data are presented in Table 2.

At the 3-year follow-up, all patients presented for evaluation at the outpatient clinic; thus, no patients were lost to follow-up. Four (36.5%) patients reported recurrence of symptoms approximately 2–3 years postoperatively, including epigastric pain and postprandial discomfort. Follow-up CT angiography demonstrated a 30–40% celiac trunk stenosis in two patients, both after stenting prior to the surgery. In the two patients who had primary laparoscopic decompression without stenting, one of them developed a recurrent 50% stenosis, while the other had soft-tissue infiltration around the celiac trunk without hemodynamically significant stenosis. Recurrent case data are summarized in Table 3. The remaining seven patients (63.6%) remained asymptomatic (five after laparoscopic decompression and two after laparoscopic decompression with adjunct endovascular treatment). Individuals who had experienced weight loss regained their weight, ranging between 1 and 37 kg (mean 8.7 ± 12.8 kg), suggesting an improvement in nutritional status. The patients’ mean BMI 3 years after surgery was 28.8 ± 7.8 kg/m^2^. All patients completed postoperative questionnaires (GIQLI and GSRS), and the collected data are summarized and presented as mean ± SD and median (IQR) in Table 4. At 3 years postoperation, patient-reported outcome measures suggested a trend toward an improvement in indigestion and pain. However, these changes were not statistically significant upon paired non-parametric analysis. These findings should be interpreted with caution due to the small sample size and consequent limited statistical power. We also estimated effect sizes r, and only the effect size of pain reduction reached moderate effect level (r = 0.407).

## 4. Discussion

MALS is a rare vascular disorder in which the median arcuate ligament externally compresses the celiac artery. Although imaging findings of celiac trunk compression are quite common, their clinical significance can vary significantly. In our cohort of 37 patients with radiologically established diagnosis, only 29.7% had symptoms consistent with hemodynamically significant MALS, requiring surgical treatment. These results are consistent with anatomical and radiological studies showing that 10–24% of individuals may have celiac axis compression without clinical manifestations [3,6]. Therefore, for an accurate diagnosis, it is essential to correlate the clinical picture with imaging data.

According to established epidemiological patterns, in our study, symptomatic patients were predominantly middle-aged women, which is consistent with many publications indicating that the main affected population is women aged 30–60 years [1,22,23]. All symptomatic patients had classic signs: epigastric pain after meals, intermittent exacerbations, and weight loss, which is consistent with the findings of the other studies [7,23,24,25]. In all cases, CT angiography confirmed a high degree of proximal celiac artery stenosis, underscoring the value of this modality for assessing vascular compression [6,22,26].

Laparoscopic median arcuate ligament decompression was successfully performed in all patients without intraoperative complications. These data confirm a growing body of evidence showing that the minimally invasive approach is safe, effective, and associated with low perioperative morbidity [2,4,27,28,29]. The duration of the surgery and the length of hospitalization in our study were similar to the previously published results [28,29], and there was only one minor postoperative complication, further underlining the favorable safety profile of laparoscopic treatment.

The role of endovascular therapy in the case of MALS is still under discussion. Two of our cohort patients underwent an unsuccessful celiac artery stenting surgery prior to surgery, which did not relieve symptoms or eliminate stenosis. This discovery is consistent with numerous case descriptions and small series, which conclude that primary stenting alone is rarely effective because the external ligament compression persists and increases the risk of restenosis [30,31,32]. Reports of angioplasty or stenting without surgical decompression describe only temporary or incomplete symptom relief [18,30,31].

Columbo et al. reported that PTA was performed on 7/21 (33%) patients because of persistent symptoms and CT observed stenosis. A majority—81% of the patients—reported early symptom improvement, and 66% were able to return to work [33]. Stenting is apparently much more important after a surgical decompression, where residual internal stenosis or changes in the arterial wall persist, despite adequate ligament release. Two of our patients needed postoperative stenting in a hybrid treatment strategy. However, both had no repeat stenosis during the follow-up period, and they had no gastrointestinal symptoms. The published literature recognizes that hybrid-therapy laparoscopic decompression combined with postoperative or intraoperative stenting can benefit individual patients [5,33,34,35]. Michalik et al. documented improved flow following staged hybrid treatment [5], and Schneider et al. demonstrated the feasibility of systematic hybrid laparoscopic endovascular approaches in a dedicated hybrid operating room [34]. After the 6-month follow-up of six patients, the primary and secondary patency of the stents reached 82 percent and 100 percent, respectively [34]. This series of cases shows that median arcuate ligament resection associated with stenting seems to be a safe and effective treatment approach for MALS. However, evidence is limited to low-level studies, case reports, and limited experience [2,18,30,31,32,33,34,35], and there is no consensus exists regarding the optimal sequencing or indications for stenting in MALS.

At present, robotic median arcuate ligament release is increasingly used for MALS, as 3D-magnified vision and wristed instruments enable precise circumferential dissection and celiac neurolysis, aligning with modern diagnostic and management algorithms [12,36]. Short-term outcomes are consistently favorable: a 23-study review (290 patients) reported ~1.4% conversion, low morbidity, and short stays (~2 days), while an institutional series showed minimal blood loss, low conversion, early symptom relief, and improved duplex hemodynamics (lower celiac PSV) [12,13,14,15,37]. Durability is generally strong but variable: one large, minimally invasive series showed ~90% pain-free at 1 year (some needed adjunct angioplasty for persistent high velocities), and a 52-patient cohort reported symptom resolutions of 96%, 92%, and 88% at 6 weeks, 1 year, and 2 years, with occasional late recurrence reported in earlier studies [13,38,39]. Comparative data indicate robotic and laparoscopic approaches are both feasible in experienced centers with similar objective outcomes. In an early single-institution comparison (12 laparoscopic vs. 4 robotic), there were no conversions or major complications, length of stay was similar (~1–2 days), and laparoscopy had shorter operative time, with symptom and narcotic-use improvements in both groups [11,15,40,41,42,43].

Published experience indicates that when median arcuate ligament release did not consistently re-establish adequate antegrade celiac/hepatic perfusion, adjunctive revascularization was employed, including pancreatic arcade reconstruction with IPDA-GDA anastomosis in the setting of pancreaticoduodenal arcade aneurysms, end-to-end middle colic artery-GDA anastomosis during pancreaticoduodenectomy, reimplantation of the left gastric artery onto the supra-celiac aorta, and aorto-hepatic bypass using a PTFE graft, including cases performed after unsuccessful endovascular intervention [44,45,46,47]. In a pressure-gradient-guided series, a persistently hemodynamically significant post-release gradient prompted escalation to PTFE bypass reconstruction, most commonly via aorto-celiac or aorto-hepatic bypass [48]. Overall, MAL release is the preferred first-line treatment, but a minority of patients require flow-directed revascularization via collateral-based anastomosis or aortic inflow bypass when perfusion or pressure gradients remain inadequate after decompression.

After median arcuate ligament release, intraoperative evaluation of arterial flow in the celiac trunk must be performed. Doppler ultrasound assessment before and after decompression can confirm whether the procedure was successful by showing improved waveforms and velocity in the celiac trunk and, more distally, in the common hepatic artery (CHA) and gastroduodenal artery (GDA) [49]. To assess arterial flow quality in cases where the Doppler does not clearly confirm whether flow is sufficient for visceral organs to function properly, transit-time flowmetry (TTFM) can effectively detect retrograde blood flow in the CHA or GDA and its recovery after decompression, allowing for the quantitative measurement of volumetric blood flow [50]. If flow remains borderline, a temporary test conduit from a high-pressure source (typically the SMA, GDA, or aorta) can be constructed. For example, an inferior pancreaticoduodenal artery–GDA, the left gastric artery to the supra-celiac aorta, or the proper hepatic artery to the aorta conduits, using a polytetrafluoroethylene graft, can be constructed to evaluate if flow after decompression becomes antegrade. Measuring flow through this conduit simulates definitive revascularization and serves as a functional stress test of the celiac vascular bed [36,48]. A significant increase in flow suggests adequate distal capacity, whereas persistently low flow indicates the need for permanent bypass [36]. In our series, we used the laparoscopic lysis of the celiac trunk and secondary angiographic evaluation postoperatively, as well as endovascular correction, if needed.

Angioplasty and stenting of the celiac artery play a significant role in a hybrid treatment approach, including decompression of the median arcuate ligament and staged revascularization as per [20]. Endovascular adjuncts are reserved for cases with residual symptoms and flow-limiting stenosis (>70%), as well as, if after release, a significant arterial wall fibrosis exists or the vessel fail to expand despite decompression [21]. There are no clear findings about timing between primary decompression surgery and adjunct endovascular therapy; however, based on the scientific literature, endovascular intervention should be considered after a period of several weeks to several months (2–8 weeks) since the primary surgical intervention. For example, in a Columbo et al. 2015 retrospective series, staged celiac artery stent placement was performed at a mean of ~49 days after surgery [33]. The primarily used endovascular modality is usually percutaneous transluminal angioplasty (PTA). However, in cases of insufficient post-PTA luminal diameter, elastic recoil, or flow-limiting dissection, balloon-expandable stent implantation is usually indicated [33,51]. Most of the published case reports on celiac artery stenting after MAL decompression surgery have used bare-metal, balloon-expandable stents because of their strong radial force to resist recoil after angioplasty and less rigidity, which is especially important in an angulated celiac ostium [35]. Medical therapy includes periprocedural anticoagulation, typically done with unfractionated heparin and post-interventional antiplatelet treatment. There is no formal guideline specifically for MALS, but many interventional reports recommend single long-term antiplatelet therapy with aspirin after PTA without stent implantation and dual antiplatelet therapy (DAPT) for 1–6 months, followed by long-term single antiplatelet therapy with aspirin after stenting [51,52]. In addition, risk factor modification and structured clinical and imaging follow-up are advised to allow for the early detection of restenosis or recurrent symptoms.

During our 3-year follow-up period, 36.5% of patients reported a recurrence of symptoms, which is consistent with other medium- and long-term studies reporting a recurrence in 15–40% of cases [24,53,54]. Our findings are in line with Woestemeier et al. [21], wherein it was reported that long-term relief of symptoms after surgical decompression was achieved in only a fraction of patients, while many experience partial or recurrent symptoms. Their study found a younger age and shorter duration of symptoms as factors predicting better performance, stressing that MALS is influenced not only by vessel compression but also by neurogenic and functional components. The 36.5% recurrence rate in our study, despite the technically successful decompression, highlights the multifunctional nature of persistent MALS symptoms.

Radiological imaging revealed persistent or recurrent stenosis of the celiac artery in three patients and the infiltration of soft tissue in one patient. These data support the theory that surgical failure may be the result of incomplete decompression, postoperative scars, remodeling of arteries, or permanent neuropathic mechanisms associated with the celiac plexus [10,16]. The importance of careful dissection and separation of nerve fibers is highlighted in technical reports and expert recommendations [10,16,55]. It should be noted that both patients who underwent treatment with preoperative stenting developed moderate recurrence of stenosis, suggesting that endovascular interventions do not always prevent recurrence and require careful monitoring.

Postoperative quality of life assessments showed an improvement in indigestion and abdominal pain, consistent with previous reports showing a significant improvement in symptoms after decompression [55,56,57]. Although overall GIQLI scores improved, this change did not achieve statistical significance, probably due to the small sample size and the multifactorial nature of gastrointestinal complaints. Persistent symptoms may also reflect co-existing functional gastrointestinal disorders, which are widely recognized among MALS patients and may affect postoperative evaluation [20].

The management of MALS is still complicated by the absence of universally accepted diagnostic or treatment guidelines. The European Society for Vascular Surgery recognizes the lack of high-quality evidence and highlights the diversity of diagnostic tests and treatment indications [6]. The current literature mainly contains case reports, case series, and small single-center cohorts [5,7,24,25,56,57,58,59]; few prospective studies; and no randomized controlled studies. Systematic reviews regularly highlight the low methodological quality, high heterogeneity, and limited statistical power of existing evidence [2,20]. As a result, definitive recommendations on optimal diagnostic protocols, standardization of surgical techniques, and time and monitoring strategies for endovascular therapy are still lacking.

Our study provides comprehensive medium-term follow-up, with no patients lost after 3 years, and uses standardized diagnostic and surgical protocols. However, limitations include the small sample size inherent to rare diseases, the single-center design, and the absence of a control group. Also, we used standard protocol for CTA with sagittal reconstructions; however, more precise evaluation of stenoses should have been done in the expiratory phase. Moreover, to better define if stenoses were hemodynamically significant, duplex ultrasound with certain velocity thresholds needed to be included in MALS diagnostic and evaluation algorithms, which was not the case in our study. Finally, we must acknowledge that postoperative symptom assessment remains partly subjective. However, our findings support laparoscopic decompression as a safe and effective first-line treatment for symptomatic MALS, consistent with current best practice. While most patients experience durable symptom improvement, recurrence remains clinically relevant, particularly in cases of recurrent stenosis. Endovascular stenting should be reserved for carefully selected patients with persistent vascular narrowing after decompression, as evidence supporting its routine use remains limited. Given the high prevalence of asymptomatic radiologic compression and the absence of standardized diagnostic or therapeutic guidelines, future multicenter prospective studies are essential to improve patient selection, optimize treatment algorithms, and develop evidence-based recommendations for managing MALS.

## 5. Conclusions

In this small single-center cohort, laparoscopic median arcuate ligament release was feasible and safe, with low morbidity and no conversions. Most patients reported symptom improvement, and 63.6% remained asymptomatic at 3-year follow-up. However, symptom recurrence occurred in 36.5%, and PROM improvements (GIQLI/GSRS) did not reach statistical significance. These findings support the procedure’s safety and potential clinical benefit in carefully selected symptomatic patients, while highlighting the need for standardized diagnostic criteria, clearer outcome definitions, and larger studies to better define the durability and the role of adjunct endovascular treatment for residual stenosis. Adjunct endovascular treatment was used in selected cases with recurrent stenosis. However, the sample size was too small to draw firm conclusions regarding its impact on long-term patency or symptoms.

## Figures and Tables

**Table 1:** Demographic and clinical characteristics of patients.

Gender	
Male	2 (18.2)
Female	9 (81.8)
Age, year	56.7 ± 10.7
Comorbidities	
Autoimmune thyroiditis	1 (9.1)
GERD	2 (18.2)
Hypertension	5 (45.4)
Depression/anxiety	3 (27.3)
BMI prior surgery, kg/m^2^	24.8 ± 7.3
BMI after surgery, kg/m^2^	26.8 ± 7.8
Clinical presentation	
Epigastric pain	11 (100)
Postprandial pain	11 (100)
Weight loss	7 (63.6)
Mean weight loss, kg	14 ± 11.6
ASA score	
I	8 (72.7)
II	3 (27.3)
Prior abdominal surgery	3 (27.3)
CT angiography of celiac trunk stenosis	
50%	6 (54.5)
70–80%	1 (9.1)
80–90%	4 (36.4)

**Table 2:** Surgical data.

Operation duration, minutes (mean ± SD)	103 ± 54
Hospital-stay, days (mean ± SD)	4.2 ± 2.2
Intraoperative complications, n (%)	0
Postoperative complications, n (%)	1 (9.1)
Celiac artery stenting prior to surgery, n (%)	2 (18.2)
Celiac artery stenting after surgery, n (%)	2 (18.2)

**Table 3:** Summarized recurrent case data.

Case	Time to Recurrence	Symptoms at Recurrence	CTA Finding at Recurrence	Prior Stent (Preop)	Postop Stent (After MAL)	Subsequent Management	Status at Last Follow-Up
1	2–3 years	Epigastric pain + postprandial discomfort	30–40% celiac trunk stenosis	Yes	No	Conservative	Persistent
2	2–3 years	Epigastric pain + postprandial discomfort	30–40% celiac trunk stenosis	Yes	No	Conservative	Persistent
3	2–3 years	Epigastric pain + postprandial discomfort	Recurrent ~50% stenosis	No	No	Conservative	Persistent
4	2–3 years	Epigastric pain + postprandial discomfort	Soft-tissue infiltration around celiac axis, no hemodynamically significant stenosis	No	No	Conservative, GI work-up, pain management	Persistent

**Table 4:** Preoperative and postoperative questionnaires. GIQLI total scores range from 0 to 144 (higher scores indicate better quality of life). GSRS domain scores range from 1 to 7 (higher scores indicate greater symptom severity).

	Before Surgery	After Surgery	*p*
GSRS Diarrhea score			
Median (IR)	1 (0.5)	1.0 (1.0)	
Mean (SD)	1.22 ± 0.44	1.44 ± 0.73	0.48
GSRS Indigestion score			
Median (IR)	2 (3.5)	1 (1)	
Mean (SD)	2.67 ± 1.8	1.78 ± 1.3	0.32
GSRS Constipation score			
Median (IR)	2 (3.5)	3 (4.5)	
Mean (SD)	2.67 ± 2.29	3.22 ± 2.28	0.48
GSRS Pain score			
Median (IR)	4 (1.5)	4 (4.0)	
Mean (SD)	4.22 ± 1.39	3.56 ± 2.03	0.22
GSRS Reflux score			
Median (IR)	1 (4)	1 (2)	
Mean (SD)	2.56 ± 2.19	2 ± 2	0.41
GIQLI score			
Median (IR)	126 (44)	130 (27)	
Mean (SD)	128.67 ± 14.77	132.11 ± 20.87	0.69

## Data Availability

The raw data supporting the conclusions of this article will be made available by the authors upon request.

## Abbreviations

The following abbreviations are used in this manuscript:

MALS	Median arcuate ligament syndrome
CT	Computed tomography
PTA	Percutaneous transarterial angioplasty
